# Exploring Theory of Mind Use in Blind Adults During Natural Communication

**DOI:** 10.1007/s10936-015-9379-x

**Published:** 2015-05-24

**Authors:** Jolanta Sak-Wernicka

**Affiliations:** Department of Modern English, The John Paul II Catholic University of Lublin, Al. Racławickie 14, 20-950 Lublin, Poland

**Keywords:** Blindness, Communication, Theory of mind, Mindreading, Adults

## Abstract

The aim of this article is to explore whether people who are blind are as successful in recognising other people’s mental states in communicative situations as people who are sighted. In the current investigation, a group of blind and sighted individuals were tested on their first and higher-order ToM abilities to recognise the intentions, feelings and beliefs of people engaged in natural conversations. The results revealed significant differences between the groups in the recognition of mental states, but no differences were found in their first-order and higher-order ToM use. The study shows that people who are blind may understand other people’s intentions, feelings and beliefs differently than people who are sighted. This is not because of their ToM deficits or linguistic incompetence, but because during communication blind individuals have limited access to the information about others’ mental states.

## Introduction

Theory of mind (ToM), generally defined as the ability to impute mental states to oneself and others (Premack and Woodruff 1978), is integral to the process of communication. Because what is normally said does not fully determine what is actually meant (Sperber and Wilson 2002), it is constantly necessary for listeners to make inferences about a speaker’s mental states in order to understand their messages, judge their behaviour, and predict their responses. This (at least partially) is possible through observing the speaker’s actions, facial expressions, gestures and other non-verbal cues. Among other things, visual cues allow an individual to recognise a conversation partner’s attitude or attentional direction; to monitor their understanding of a message (Clark and Krych 2004), or to surrender the floor when the partner wishes to take a turn (Kendon 1967; Agryle 1973; Bavelas et al. 2002). It appears clear that vision has the power to provide important information about people’s mental states, but whether the lack of visual cues impairs ToM is far from obvious.

One may expect that blind people who are deprived of visual information and can only rely on linguistic experience, will be less successful in inferring other people’s communicative intentions. In other words, the information which people with visual impairments (VI) have access to and which should allow them to attribute mental states to their conversation partners, is different from the information accessible to people with normal vision. Hence, it appears well-justified to ask whether the amount of information accessible to blind people during daily communication allows them to successfully “read other people’s minds”. Without knowing whether linguistic information can effectively compensate for the missing visual cues, it is not possible to ascertain whether people who are blind should have visual information verbally provided to them during communication in order to improve their understanding of messages. Therefore, the aim of this article is to explore ToM use in blind people and to investigate whether these individuals, if provided with no visual information, can be as successful in recognising other people’s mental states in communicative situations as people who are sighted.

For many years it has been argued that vision plays a critical role in ToM development and children with VI are significantly delayed in acquiring ToM abilities (Minter et al. 1998; Sonksen and Dale 2002; Korkmaz 2011). Studies using modified ToM tasks based on auditory and/or tactile experience (see Table 1 for more detailed descriptions of the tasks), showed that children who were blind were less successful than their peers with normal vision (e.g. Brambring and Asbrock 2010; Green et al. 2004; McAlpine and Moore 1995; Minter et al., ibid.; Peterson et al. 2000). Researchers explained that the poor performance of blind children was due to the fact that the individuals had little experience in social interactions and they had not learnt about other people’s mental states by observing their facial expressions, gestures and other non-verbal behaviour. At the same time the studies indicated that it is much more difficult to attribute mental states to others on the basis of verbal information only. Therefore, it was concluded that by missing out visual cues blind children miss out on an important (pragmatic) part of a conversation which enable sighted individuals to resolve “the discrepancy between what is actually understood about the experience of another person and what would be expected on the basis of verbal competence” (Dyck et al. 2004, p. 791). For the above-mentioned reason, ToM deficits in blind individuals are often argued to affect their pragmatic language abilities of interpreting intended meanings of people’s utterances (see e.g. James and Stojanovik 2007; Tadić et al. 2010).

**Table 1:** ToM tests used with blind individuals

Type of task	Example task	Procedure
*Auditory*		
Story-based	False-belief stories (Pijnacker et al. 2012; based on Sally-Ann Task by Wimmer and Perner 1983)	Participants listen to stories where an object changed location in the absence of another person and are asked where this person will look for the object
	Non-literal stories (Pijnacker et al. 2012; based on Strange Stories Test by Happé 1994)	Participants listen to stories which include different figures of speech and they are asked to explain what characters of the story meant by what they said
Activity-based	Unexpected outcome task (Brambring and Asbrock 2010)	Participants listen to series of related sounds. They are asked to predict the next sound and an unexpected sound is played. They are asked what they thought the sound would be and what someone else would think
*Tactile*	Unexpected content task (McAlpine and Moore 1995; Peterson et al. 2000; based on Deceptive Box Test by Perner et al. 1987)	Participants are given to explore objects with some unexpected content (e.g. an egg carton with balls, a teapot with sand, a hamburger box with a sock). They are asked what they thought was inside and what someone else would say about the content of the object
	Unexpected outcome task (Brambring and Asbrock 2010)	Participants explore boxes which contain objects in predictable sequence, except for the last box the content of which is different. They are asked what they thought was in the last box and what someone else would say about the contents of the last box
	Unexpected dislocation (Brambring and Asbrock 2010; based on Sally-Ann Task by Wimmer and Perner 1983)	A participant puts a coin in a box, wallet or basket in the presence of a family member. The family member leaves and the participant with an experimenter move the coin to another container. The participant is asked where the family member will look for the coin

Only recently, however, it has been suggested that vision may not be necessary for the development of ToM, and that congenital blindness does not have to affect mindreading. Recent studies show that the delays in the development of ToM are more specific to children whose blindness is linked to neural causes (ocular-plus blindness), and that the performances of children with ocular blindness in relevant ToM tasks do not differ from sighted children (Beeger et al. 2014). Similarly, a study performed on children with a broad range of VI (including low vision) demonstrates no deficits in the participants’ ToM or pragmatic language abilities (Pijnacker et al. 2012). This indicates that ToM development may not depend on visual experience. Finally, fMRI studies have revealed that ToM in congenitally blind and in sighted adults is selectively localised in the same brain regions, which include the bilateral temporoparietal junction, medial prefrontal cortex, precuneus and anterior temporal sulci (Bedny et al. 2009). The findings show that blindness does not change the ToM network and blind individuals develop ToM independently of visual experience. This suggests that blind people are mentally equipped to recognise other people’s thoughts, feelings and intentions, but it remains unknown whether the information they are provided with during a conversation allows them to be as successful in mindreading as people who are sighted.

Blind individuals learn about mental states from linguistic experience and they use this source to infer what others think, feel or believe during communication. Although it is true that a substantial amount of information can be provided not only by what people say but also how they say it, no studies have been performed to reveal how accurate blind people’s inferences are about others’ mental states. Because the sense of sight carries different information than hearing, it is possible that having no access to visual information or to the descriptions of what others can see, a blind individual may be unable to adequately recognise the emotional content of a speaker’s utterance and, for example, to distinguish between when the speaker is angry and when she or he just pretends to be so. Also, without the access to visual cues, it may not be possible for the blind person to follow other people’s actions and predict their behaviour. This is in comparison to a person who has unlimited access to both visual and auditory information during communication. Because presently there are hardly any tests which are designed to detect ToM difficulties in real communicative situations, it is impossible to determine whether blind people are less successful in reading the minds of their communication partners than sighted people, and whether their visual limitations may affect their performance.

Until now ToM in typically developing children and normal adults has been examined via the use of different tasks (see Table 2 for review) which were developed to explore different ToM mechanisms. These tasks were based on either the observation of situations and pictures, or listening to short stories, and inferring characters’ mental states from their verbal or non-verbal behaviour. Unfortunately, these methods of testing ToM are more often claimed to provide unreliable results. Among other things, this is because many of them put a considerable cognitive load and require additional abilities other than ToM (Bloom and German 2000). For example, in the standard Sally-Ann Task, participants are expected to not only put considerable effort into remembering the details of the situation, but also reason about a belief which is false. This is said to be much harder than recognising other people’s true beliefs.

**Table 2:** The review of ToM tests

Type of task	Example task	Target group	Procedure
Situation-/picture-based	Sally-Ann Task (Wimmer and Perner 1983)	Children	Standard false-belief task; participants observe two characters and infer where a doll (Sally) will look for an object which it put in one place before going away, and which another doll (Ann) has moved to a different place in Sally’s absence
	Deceptive Box Test (Perner et al. 1987)	Children	Participants are shown a Smarties tube and they are asked what they think is inside. When they recover that there is something else than what they have expected, they are asked what another person (not having looked inside) would think the tube contains
	Reading the Mind in the Eyes Task (Baron-Cohen et al. 1997)	Adults	Participants read emotions from the photographs in which different pairs of eyes are presented.
	Character Intention Task (Sarfati et al. 1997)	Both	Participants predict intentions of characters whose actions are presented in comic strips
Text-based	Strange Stories Test (Happé 1994)	Both	Participants are given short stories which include different figures of speech. The participants infer what characters of the story meant by using metaphor, irony, sarcasm etc.
	Faux Pas Recognition Test (Stone et al. 1998; Baron-Cohen et al. 1999)	Both	Participants recover a faux pas made by one of characters in a short story
Mixed	The Awareness of Social Inference Test (McDonald et al. 2006)	Adults	The test involves the interpretation of emotional displays and sarcasm from vignettes
	The New ToM Test (Muris et al. 1999)	Children	Vignettes, stories and drawings are used to test first-, second- and advanced ToM

Secondly, many of these tests use artificial pre-scripted scenarios or static, decontextualised pictures which can hardly be compared to a dynamic, real-life situation. It is worth mentioning that in text-based tasks the context is provided through verbal descriptions of situations and characters. This does not happen during real-life communication when listeners themselves have to make proper assumptions about speakers’ mental states based on the contextual cues which are available to them. Additionally, as observed by Byom and Mutlu (2013, p. 3), the available tests “do not require individuals to formulate appropriate response as if they themselves were in the situation.” This is because they consider only one answer correct and fail to account for the fact that there may be subtle differences in individual people’s interpretations of a speaker’s verbal or non-verbal behaviour. The differences, however, do not have to indicate ToM deficits, because during natural communication sharing exactly the same thoughts, feelings and intentions between partners does not take place either.

Despite the growing interest into the effect of blindness on ToM, there are still not many tests that would be properly adapted or designed to meet the needs of blind people. This mainly concerns adults whose ToM abilities, when compared to children, remain seriously underexplored. Because the majority of studies in the blind population have been performed with the intention of examining first-order ToM (i.e. attributing thought), there is a significant shortage of tests which could be used with adults to examine higher-order ToM abilities (i.e. attributing thought about thought). Also, there are hardly any tests aimed to compare human abilities to recognise mental states other than beliefs.

The main objective of this article is to explore whether people who are blind are as successful in taking other people’s perspective as sighted people and whether they are able to recognise speakers’ mental states in communicative situations when compared to people who are sighted. The article reports an exploratory study performed on a group of blind and sighted adults who were tested on their representational (first-order) and meta-representational (higher-order) ToM abilities to make inferences about intentions, emotions and beliefs of others.

## Methods

### Participants

A total group of 39 adults between 19 and 67 years of age participated in the study. There were 19 adults with no functional vision (8 women and 11 men) who were recruited from the Polish Association of the Blind, Blind Co-operative Society and Occupational Therapy Workshops. The participants were congenitally blind or early-blind (i.e. they lost sight no later than at the age of 5). Individuals with ocular-plus blindness, partial vision and who were late-blind did not take part in the study. 20 participants with normal vision (10 women and 10 men) were recruited from the John Paul II Catholic University of Lublin and also participated in the study. The participants were students and administrative/technical workers at the university. Participant characteristics are shown in Table 3.

**Table 3:** Participant characteristics

	Blind ($\hbox {n} = 19$)	Sighted ($\hbox {n} = 20$)
*Gender*		
Male	8	10
Female	11	10
*Age range*		
19–25 years	6	6
26-35 years	3	5
36-45 years	4	1
46-67 years	6	8
Mean (SD)	38.32 (19.00)	38.55 (15.60)
*Education*		
Primary	4	0
Secondary	10	12
Higher (BA degree)	1	2
Higher (MA degree)	4	6

All participants were Polish native speakers. The participation in the study was voluntary and all participants gave informed consent. The study was approved by the institutional review board of the John Paul II Catholic University of Lublin. It was also permitted by the president, director and psychologist of the organisations in which blind participants were tested.

### Material and Procedure

The study was designed to explore blind people’s ToM abilities during natural communication. For this purpose, all participants of the study were presented with 12 short dialogues based on real life conversations overheard at bus stops, in supermarkets, on television, in offices or restaurants. Six dialogues were chosen to test the understanding of first-order ToM (i.e the ability to attribute mental states to a person) and in six others higher-order ToM (i.e attributing a person’s mental states about another person’s mental states, understanding figurative language) was examined. Additionally, the recognition of different mental states (intentions, beliefs and emotions/feelings) was tested in both first-order and higher-order dialogues. All dialogues took place between no more than two people and varied in exact length between 7 and 32 s. The dialogues were performed by professional actors and recorded using a digital HD camera. Next, the soundtrack from the film was separately recorded.

In order to determine whether blind participants understood other people’s mental states during communication compared to sighted participants, they were asked to listen to the recording with the dialogues. Because the main intention behind this experiment was to obtain the effect similar to real life situations, the participants were provided with no additional information or descriptions of what was happening in the situations, and they were expected to make proper inferences on the basis of available contextual cues. Also for the above-mentioned reasons, the participants were not informed about the purpose of the study until the experiment was over. After listening to each dialogue, the participants were asked a question about one of the speakers’ intentions, emotions/feelings or beliefs.

In order to help the participants verbalise their ideas and facilitate the analysis of their responses, they were given a questionnaire with closed questions. The questionnaire contained a few possible options to choose from. Among the suggested options, only one answer was correct and the participants were specifically instructed to choose only one answer in response to each question which they felt best suited their own interpretation. If the participants were unable to formulate their own responses, they were asked to choose the option ‘I don’t know’. If they thought that none of the provided options was appropriate, they could also offer their own individual answers. The responses were then analysed by the experimenter and they fell into the same categories as the suggested options (correct or incorrect), but were expressed subjectively. Participants’ responses were considered incorrect if they avoided providing explicit answers (e.g. parroted utterances used in the dialogues, recapitulated the main points of the conversation), or if they provided far-fetched or incoherent responses based on faulty assumptions. For each dialogue the participants could receive 1 or 0 points. The questionnaire was appropriately adapted in braille and an electronic version was available to users of braille displayers.

The blind participants’ performance was compared to sighted participants who were provided with the same dialogues, but presented as film clips. They were asked to complete the same questionnaire by choosing one of the provided answers or offering their individual responses. The examples of the dialogues, testing questions and answer choices which were used in the study are presented in Table 4.

**Table 4:** Examples of dialogues (with different mental states), testing questions and answer choices

Theory of mind	Dialogue	Question	Answers (correct in bold)
First-order (intention)	A: I have two tickets for “Don Giovanni.”	The woman intends to:	a. boast about the tickets which she has managed to get hold of
	B: Who’s he fighting with?		**b. invite the man to the opera**
	A: It’s an opera.		c. tell the man that she has bought tickets for the opera which they both wanted to see
	B: Shall we meet later?		d. other answer (if so, suggest what)
			e. I don’t know
(belief)	A: When did it happen?	The woman thinks:	**a. it will be difficult to remove the wine stain**
	B: About an hour ago.		b. it is too late to find any shop open and buy the wine
	A: What kind of wine?		c. they can discuss the other woman’s problems
	B: Burgundy.		d. other answer (if so, suggest what)
	A: That’s ok. We can deal with it. Is it cotton and silk?		e. I don’t know
(feeling/emotion)	A: Honey, which bathroom tiles do you like more: the green ones or the ones with the subtle border?	The woman feels:	a. surprised that her husband shares her taste and that they’ve chosen the same tiles
	B: The ones with the border are ok.		b. confused about which tiles to choose and hopes her husband will help her make this choice
	A: Don’t you think that the border is too subtle.		**c. determined to choose the green tiles and she tries to suggest her choice to her husband**
	B: The other ones, then.		d. other answer (if so, suggest what)
	A: I like them more, too. You see how well we understand each other.		e. I don’t know
Advanced (intention)	A: What do you have there?	The woman thinks the man has a problem with:	a. playing a song
	B: I’ve bought Sting’s new single.		**b. opening the CD**
	A: Let’s hear it. (after a short while)		c. nothing
	B: Pull the tab!		d. other answer (if so, suggest what)
			e. I don’t know
(belief)	A: We need a new bedspread ... and carpet ...	The man thinks:	a. the children will not approve of the exchange of old things for new ones
	B: If a carpet, then chairs, if chairs, then a mirror, wardrobe and then our kids will hate us.		b. the choice of new things will lead to conflict between him and his wife
	A: Why?		**c. he and his wife can’t afford to buy new things**
	B: Because we will divorce.		d. other answer (if so, suggest what)
			e. I don’t know
(feeling/emotion)	A: Have you called the neighbour to apologise to her?	Justine wants to say that she is:	a. angry that the neighbour offended Bart during a phone call
	B: Just like you told me.		**b. surprised that apologizing to the neighbour was so difficult to Bart**
	A: And did your world come crashing down?		c. sorry that something tragic happened to Bart
			d. other answer (if so, suggest what)
			e. I don’t know

## Results

In order to ascertain whether the blind and sighted groups differed in their attribution of mental states, the total scores in both groups for all dialogues were calculated. Prior to performing statistical analyses preliminary assumption testing was conducted, and no violations to the assumptions of normality (The Shapiro–Wilk: $F = .93, p = .16; F = .94, p = .28$) or equality of variances [The Levene’s test: $F(2,37) = 1.32, p = .25$] were found. Overall, both groups performed slightly above chance level providing 62 % (sighted participants) and 51 % (blind participants) of correct responses. Only, 5 % of blind and sighted participants failed to provide any answer. Next, an independent-samples $t$ test was conduced to compare the scores in both groups. The analysis revealed significant differences between the group of blind (M $=$ 6.15, SD $=$ 2.26) and the group of sighted participants (M $=$ 7.5, SD $=$ 1.82); t(37) $=$ 2.04, $p$ $=$ .048.

In order to find out whether the statistically significant differences were due to the blind participants’ inability to reason about speakers’ mental states at a higher level, the sum scores were separately calculated for the dialogues with first-order and higher-order ToM (see Table 5 for the mean scores). The scores were entered into an ANOVA with group (blind, sighted) as a between-subject factor and ToM-level (first-order, higher-order) as a within-subject factor. The analysis revealed no significant interaction between groups and ToM-level [$F(1,74) = .01, p = .91$]. There was also no main effect for ToM-level [$F(1,74) = .58, p = .45$], but the main effect for group was significant [$F(1,74) = 4.94, p = .029$].

**Table 5:** Mean scores for blind and sighted groups (ToM-levels)

	Mean (SD)	
	Blind	Sighted
First-order ToM	3.21 (1.13)	3.85 (1.03)
Advanced ToM	2.94 (1.80)	3.65 (1.22)

In the analysis we also wanted to examine whether the previously mentioned statistical differences between the blind and sighted groups were caused by discrepancies in the understanding of mental states made manifest by speakers in the dialogues. By doing this, we hoped to find out whether some mental states are more difficult to recognise during communication than others. For these purposes, the sum scores for the dialogues with intentions, emotions/feelings and beliefs were calculated (see Table 6 for mean scores in the groups), and entered into an ANOVA with group (blind, sighted) as a between-subject factor and mental state (intention, feeling, belief) as a within-subject factor. No interaction between group and mental state was found [$F(2,111) = 1.05, p = .35$], but there were significant main effects for group [$F(1,111) = 6.89, p = .01$] and for mental state [$F(2,111) = 6.09, p = .003$]. Post-hoc comparisons using the Tukey’s HSD test indicated that the scores for dialogues with beliefs were significantly different from the dialogues with feelings ($p$ $=$ .004) and intentions ($p$ $=$ .022).

**Table 6:** Mean scores for blind and sighted groups (mental states)

	Mean (SD)	
	Blind	Sighted
Intentions	2.26 (.99)	2.8 (.76)
Beliefs	1.78 (1.03)	1.9 (.71)
Emotions/feelings	2.05 (1.02)	2.75 (.96)

## Discussion

The aim of this study was to explore whether the lack of vision may impact blind people’s ToM during communication. More specifically, it was intended to examine whether the information people with VI are normally provided with in different communicative situations allows them to recognise other people’s intentions, feelings and beliefs compared to people who are sighted. To accomplish this goal, the two groups of blind and sighted participants were tested on their abilities to attribute different mental states to speakers engaged in conversations which were real life situations chosen to fulfil the purpose of this experiment.

The first significant finding of the study was that the blind and sighted groups differed in how successful they were in ToM use. The comparison of the total scores obtained by the blind and sighted participants indicates that vision helps to recognise other people’s mental states, and provides more accurate information about the contents of others’ minds than language alone. This may suggest that linguistic experience may not always compensate for the missing visual cues, and in specific contexts may turn out to be an insufficient source of information. Having no access to visual cues, people may find it much harder to make proper inferences about others’ mental states and to understand their intentions, thoughts and feelings, as it is often the case during a phone conversation. Although the individuals will be able to interpret a verbal message by making some inferences about people’s mental states from their pitch, tone of voice or other prosodic features of speech, their interpretations may still differ from what they would understand, if they could observe speakers’ facial expressions, gestures and other non-verbal cues. For this reason, in some situations it may be necessary to provide blind people with relevant information they cannot access. Such attempts have been already made by introducing audio-description in media and by providing blind individuals with recorded information about the appearance or non-verbal behaviour of film characters. Much less popular is live audio-description which provides the information in real-time during lectures, football matches or informal meetings. The findings of the present study suggest that it may be vital that audio-description become part of natural communication, especially when the information blind individuals cannot access and easily infer from what people say is crucial for the interpretation of messages.

As indicated above, the study also revealed the differences between the blind and sighted groups in the recognition of specific mental states. Overall for both groups, inferring what speakers’ thought was more difficult than inferring what they intended to do or what they felt on a given occasion. Although this issue calls for further and more detailed research, it appears that recognising people’s beliefs requires reaching deeper into the contents of their minds than recognising their emotions or intentions which are more ostensively manifested. As a result, one can conclude that mental states vary in difficulty and require different degrees of effort. It is also possible that attributing thoughts to others operates on a different level of ToM than attributing other mental states. Accordingly, people’s success in mindreading may depend on what mental states they are expected to recognise in others. Because previous ToM research has mainly involved the recognition of other people’s beliefs, which (compared to other mental states) may be the most difficult to recognise, more attention should be paid to intentions, feelings and emotions in future studies.

Despite significant differences in attributing particular mental states, the performances of the groups participating in the study did not differ in the dialogues with first-order and higher-order ToM. This was regardless of whether the participants had to mentalise about thoughts, feelings and intentions of speakers, identify what mental states the speakers attributed to others, or what they wanted to communicate using figurative language. This indicates that both blind and sighted participants have fully developed ToM and they are able to make use of this ability to interpret utterances in different communicative situations. It also appears that any differences in the understanding of other people’s mental states between blind and sighted individuals result only from perceptual limitations of the blind participants and their inability to observe on-going communicative situations. The differences are not related to developmental deficits which would impair advanced ToM abilities.

Although in the study both groups were found perfectly able to make full use of their ToM abilities, they did not perform as successfully as it might have been expected. This surprising finding led us to the conclusion that during natural communication people may not read that much into the intentions, beliefs and emotions of others, and they may concentrate more on the general meaning of a message. Apart from the fact that individual people may be more, or less predisposed to reflect on the mental states of others, they may avoid mentalising about possible, (hidden or overt) mental states of speakers and save their cognitive effort during communication. It is possible that the individuals analyse a speaker’s behaviour more carefully only when it is necessary, for example, when the speaker’s words are confusing or ambiguous. This may be in accordance to the previous studies by Keysar et al. (2003) who have observed that ToM abilities of adults are not fully incorporated into their comprehension. Although the individuals are adequately equipped and fully able to use them, most of the time they keep ToM ‘in the box’, saving it for other, perhaps more challenging tasks.

Another explanation may be that the signal people receive during communication is too complex to single out one specific thought, intention or feeling a speaker makes manifest. Therefore, any answer the participants thought they could provide might seem incomplete for them. It is also important to remember that despite our efforts to make tasks used in the study as similar to real-life situations as possible, they were not identical to natural communication. This means that the participants might not have obtained as rich contextual information as they would normally get from conversation partners, which could have had an effect on their performance and obtained results.
